# Advancements in the Pathogenesis, Diagnosis, and Therapeutic Implications of Intestinal Bacteria

**DOI:** 10.3390/cimb47020106

**Published:** 2025-02-08

**Authors:** Duofei Lu, Xianxiong Ma, Kaixiong Tao, Hongwei Lei

**Affiliations:** Department of Gastrointestinal Surgery, Union Hospital, Tongji Medical College, Huazhong University of Science and Technology, Wuhan 430022, China; luduofei@163.com (D.L.); ma_xianxiong@hust.edu.cn (X.M.)

**Keywords:** intestinal bacteria, inflammatory bowel disease, colorectal cancer, gastric cancer, irritable bowel syndrome, fecal microbiota transplantation

## Abstract

Intestinal bacteria form one of the most complex microbial communities in the human body, playing a crucial role in maintaining host health and contributing to the development of various diseases. Here, we provide a comprehensive overview of the composition and function of intestinal bacteria, the factors affecting their homeostasis, and their association and mechanisms with a range of diseases (e.g., inflammatory bowel diseases, colorectal cancer, metabolic diseases). Additionally, their advanced potential in disease diagnosis and treatment is highlighted. Therapies, such as chemotherapy, radiotherapy, and immunotherapy, are significantly impacted by intestinal bacteria, with research indicating that bacteria can enhance chemoimmunotherapy efficiency by affecting T cell recruitment and immune cell infiltration. Fecal microbiota transplantation has emerged as a promising option for treating recurrent *Clostridium difficile* infections and certain metabolic and neurological disorders. Gut bacteria-related serum metabolites serve as non-invasive indicators for diagnosing CRC, while fecal immunochemical tests offer promising applications in CRC screening. Future research is needed to better understand the causal relationships between intestinal bacteria and diseases, develop more precise diagnostic tools, and evaluate the effectiveness and safety of microbiome-targeted therapies in clinical treatment. This study provides deeper insights into the role of intestinal bacteria in human health and disease, providing a scientific basis for innovative therapeutic strategies that have the potential to transform the landscape of healthcare.

## 1. Introduction

The human intestinal bacteria constitute a complex community that coexists with the host, playing a critical role in numerous physiological processes and contributing to a variety of multifactorial diseases. The human and their symbiotic bacteria together form a “holobiont” with their combined genome, the “hologenome”, playing a key role in health and disease. Any changes in either the host or bacteria genome can lead to significant variations, even disease [1]. The gastrointestinal tract houses the richest and most diverse population of bacteria in the human body, with these microbes evolving to maintain a dynamic balance that supports normal physiological functions. These bacteria are crucial for human health, influencing our nutritional status.

Recent studies have shown that the disruptions in the delicate balance between the host and intestinal bacteria may be linked to a wide range of diseases, including metabolic disorders, chronic intestinal infections, cancer, and conditions affecting other systems and organs. In recent decades, several pathogenic mechanisms related to intestinal bacteria have been proposed, including immune response, oxidative stress, inflammation, genotoxins, intestinal barrier damage, metabolites, and tumor microenvironment (TME), which have significant scientific and clinical implications.

**Figure 1:**
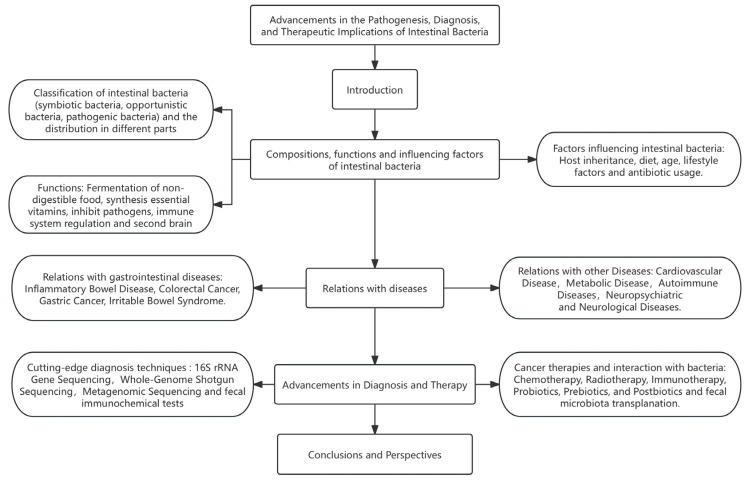
The flow chart of the literature search process.

In this review, we summarized the role of intestinal bacteria in human health, examined molecular pathogenic mechanisms of holobiont in diseases, and how microorganisms or their secreted bioactive metabolites are harnessed as potential therapeutic tools. We included studies relevant to the content and ensured that over 50% of the references were from the last five years. Studies with warning labels or questionable quality were excluded (Figure 1). This review aims to provide physicians, researchers, patients, and general health providers with a comprehensive understanding of intestinal bacteria, highlighting recent advances, limitations, challenges, and future prospects in this rapidly evolving field of research.

## 2. Intestinal Bacteria Composition, Functions, and Influencing Factors (Figure 2)

The small intestine harbors trillions of bacteria, with their concentration peaking in the lower gastrointestinal tract. The colon is dominated by anaerobic bacteria, including millions of genes mainly from phyla such as *Firmicutes* (*Ruminococcaceae* and *Lachnospiraceae*), *Bacteroidetes*, *Actinobacteria*, *Proteobacteria*, *Verrucomicrobacteria* (*Akkermannia* spp.), *Fusobacteria*, and *Cynobacteria*.

**Figure 2:**
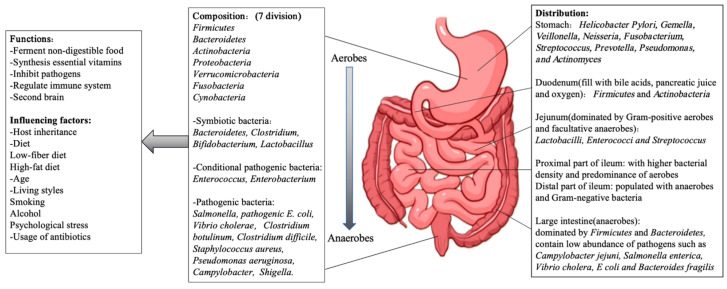
The compositions, distribution, functions, and influencing factors of human intestinal bacteria. The intestinal bacteria can be divided into 7 types, including *Firmicutes*, *Bacteroidetes*, *Antinobacteria*, *Proteobacteria*, *Verrucomicrobacteria*, *Fusobacteria,* and *Cynobacteria*. They also can be divided by the pathogenic ability of human body, such as symbiotic bacteria, conditional pathogenic bacteria, and pathogenic bacteria. Diverse bacteria have multiple functions in keeping the balance and distribution in gastrointestinal tracts, and they could be influenced by plenty of factors.

### 2.1. Intestinal Bacteria Composition

Symbiotic bacteria are microorganisms that live together and depend on each other. They are often beneficial to the host, even indispensable [2]. The majority of intestinal bacteria (over 99%) are symbiotic, including species from *Bacteroidetes*, *Clostridium*, *Bifidobacterium*, and *Lactobacillus. Bifidobacterium* and *Lactobacillus* are particularly known as probiotics, often supported by prebiotics or probiotic supplements. These bacteria aid in digestion and protect the intestines, maintaining a cooperative relationship with the host.

Conditional pathogenic bacteria are microorganisms that do not cause disease under normal circumstances but can cause disease under specific conditions (such as decreased host immunity, imbalance of flora, environmental changes, etc.). These bacteria, such as *Enterococcus* and *Enterobacterium* [3], are typically kept in check by symbiotic bacteria. However, if the balance is disrupted, conditioned pathogens can proliferate and contribute to various diseases, including cardiovascular, respiratory, neuropathic, and autoimmune diseases.

Pathogenic bacteria are microorganisms that can cause host diseases, which have strong pathogenicity and can cause diseases in normal hosts, including Salmonella [4], pathogenic *E. coli* [3], *Vibrio cholerae* [5], *Clostridium botulinum* [6], *Clostridium difficile* [7], *Staphylococcus aureus* [8], *Pseudomonas aeruginosa* [9], *Campylobacter* [10], and *Shigella* [11], and are not naturally present in intestines. Once introduced, they can disrupt the holobiont’s original homeostasis, leading to severe infections, diarrhea, food poisoning, endotoxemia, and other severe health issues.

Bacteria distribution by location: The discovery of *Helicobacter Pylori* in the stomach challenged the belief that the stomach was a “sterile organ”. Molecular techniques have elucidated the diverse bacterial communities in the stomach [12]. Factors like *Helicobacter pylori* infection, long-term antibiotic use, and proton pump inhibitors (PPIs) can alter the stomach’s bacterial composition [13]. In the small intestine (SI), the microenvironment varies across its sections. The duodenum with bile acids and pancreatic secretions supports limited bacterial density dominated by *Firmicutes* and *Actinobacteria*. The jejunum, with increased oxygen, primarily hosts Gram-positive aerobes and facultative anaerobes like *Lactobacilli*, *Enterococci,* and *Streptococci* [14]. In the ileum, particularly near the ileocecal valve, bacterial density rises with anaerobes, and Gram-negative organisms becoming more prominent, similar to those found in the colon. The large intestine (LI), by contrast, is rich in anaerobic bacteria (about 100–1000 times) more than the SI [15]. Firmicutes and Bacteroidetes dominate, and their ratio varies with age and health status, serving as an important marker for disease prediction. The bacterial composition of the LI differs significantly from the stomach and SI, with species like *Ruminococcaceae* and *Bacteroides* being more prevalent. Pathogens such as *Campylobacter jejuni, Salmonella enterica, Vibrio cholera, E coli,* and *Bacteroides fragilis* may also be present in low abundance.

### 2.2. Intestinal Bacteria Functions

#### 2.2.1. Fermentation of Non-Digestible Food

Dietary fiber, comprising indigestible carbohydrates and lignin, cannot be digested by human enzymes and relies on fermentation by large intestine (LI) bacteria. This process produces short-chain fatty acids (SCFAs) that activate G protein-coupled receptors (GPR109a, GPR41, GPR43), stimulating intestinal endocrine cell differentiation, hormone production, and appetite reduction [16]. This function helps prevent diseases like obesity, diabetes, and hyperlipidemia and strengthens the intestinal barrier. Conversely, high-fat or low-fiber diets can disrupt this balance [17], underscoring the role of dietary fiber fermentation by bacteria in maintaining LI.

#### 2.2.2. Synthesis Essential Vitamins

Humans rely on diet or symbiotic bacteria in the gastrointestinal (GI) tract for most vitamins. Bacteria such as *Lactobacillus* and *Bifidobacterium* [18] synthesize seven of the eight B vitamins (B1, B2, B3, B5, B6, B9, and B12) [19], with *Lactobacillus* vital for B12 production and *Bifidobacterium* a primary source of folate, crucial for metabolism and DNA repair. Other bacteria, including *Bacteroides fragilis* and *Eubacterium tardus*, contribute to vitamin K synthesis, aiding in disease prevention [20].

#### 2.2.3. Inhibit Pathogens

Intestinal bacteria protect the intestinal lining by competing with pathogens for nutrients and space, forming a defense line that maintains epithelial integrity and prevents pathogens from entering the bloodstream. This “colonization resistance” is critical for a balanced microbiome. *Bifidobacterium* inhibits pathogen adhesion via metabolites like teichoic acid, while *Lactobacillus* reduces intestinal pH and promotes peristalsis to expel pathogens [21]. SCFAs and bacterial antitoxins reduce intestinal permeability, decreasing metabolic disorders and pathogen invasion through anti-inflammatory mechanisms that modulate cytokine production (IL-10, IL-18). Pathogens may evolve various strategies to evade bacterial suppression, while intestinal bacteria counteract pathogen invasion through adaptive evolution and genetic mutations. Studies have shown that gut microbes evolve within the host, retaining beneficial mutations through mechanisms like single nucleotide polymorphisms, gene transpositions, and copy number variations [22]. By regulating metabolites production, gut bacteria maintain an intestinal PH that inhibits pathogen growth [23].

#### 2.2.4. Immune System Regulation

Intestinal bacteria are integral to immune development, promoting Treg cell proliferation and migration, particularly in the intestine and colon. Species like *Bacteroides fragilis* enhance anti-inflammatory cytokine IL-10 production through TLR2 activation, while *Clostridium difficile* and other bacteria produce SCFAs and tryptophan derivatives that modulate Treg cells via epigenetic pathways [24], supporting intestinal equilibrium. Additionally, *Akkermansia muciniphila* reduces inflammatory markers in colitis, indicating its role in immune homeostasis and protection against inflammation [25].

#### 2.2.5. Second Brain

The enteric nervous system (ENS, second brain) encompasses millions of neurons regulating GI functions and interacting with the peripheral nervous system (PNS), central nervous system (CNS), and intestinal bacteria [26]. This bidirectional communication, known as the gut–brain axis (GBA), influences neurological and immune responses. SCFA production and serotonin release from enterochromaffin cells affect memory, learning, and mood, with intestinal dysbiosis linked to mood disorders [27]. Probiotics can modulate the hypothalamic–pituitary–adrenal (HPA) axis, reducing cortisol levels and anxiety-related responses [28].

### 2.3. Factors Influencing Intestinal Bacteria

#### 2.3.1. Host Inheritance

Genetics partially shapes the composition of an individual’s intestinal microbiota, with studies estimating that genetics account for 1.9% to 8.1% of microbiome variability. Analysis of metagenomic sequences from 7738 participants in the Netherlands highlighted associations between certain genetic loci (LCT, ABO) [29] and specific bacterial species (*B. adolescentis*, *B. bifidum*, *C. aerofaciens*) and metabolic pathways. The correlation between the LCT locus and Bifidobacterium is particularly robust and consistent across studies [30,31]. Beyond genetics, factors such as age, sex, ethnicity, BMI, and sample handling also contribute to microbiome diversity.

#### 2.3.2. Diet

Diet strongly influences intestinal microbiota, with dietary nutrients directly affecting bacterial growth and indirectly shaping microbiota through metabolism and immune function. Fiber-rich diets promote bacteria like *Bacteroides, Bifidobacterium*, and *Ruminococcus* that metabolize complex carbohydrates, while high-fat diets increase bacteria producing lipopolysaccharides (LPS) [32]. For example, saturated fats promote bacteria associated with weight gain and insulin resistance, whereas unsaturated fats support beneficial bacteria (*Bifidobacterium, Akkermansia* and *Lactobacillus*) [33]. High-fat diets increase bile-tolerant bacteria (*Alistipes* and *Bilophila*) [34], while plant-based diets foster bacteria like *Roseburia* and *E. rectale*, which metabolize fiber. Studies comparing African children (high-fiber diets) and European children (high-fat diets) reveal notable differences in bacterial composition, such as a higher Bacteroidetes/Firmicutes ratio in African children [35].

#### 2.3.3. Age

Intestinal microbiota diversity evolves over the human lifespan. Infants initially have low diversity dominated by *Bifidobacterium* and *Lactobacillus*, with diversity increasing after solid foods are introduced and peaking in early adulthood. During adolescence, the intestinal microbiota is typically stable and diverse but may be influenced by hormonal fluctuations. In adulthood, changes in the relative abundance of *Akkermansia muciniphila* may be linked to the host’s metabolic health, and its relative abundance tends to decrease over time [36]. From around age 65, diversity generally declines [37]. Studies show that long-lived individuals have high diversity, particularly in beneficial families like *Lachnospiraceae* and *Ruminococcaceae*, suggesting a link between microbial richness and longevity [36].

#### 2.3.4. Lifestyle Factors

Lifestyle choices impact intestinal microbiota balance. Smoking, excessive drinking, stress, poor hygiene, and lack of sleep or exercise all alter microbial diversity. Smoking increases gut pH and bile acid metabolites, promoting bacteria like *Eggerthella* [38] and *Lactobacillus* while reducing *Bifidobacterium* [39]. Alcohol use decreases beneficial bacteria (*Roseburia*, *Faecalibacterium*) and increases pathogen-associated bacteria (*Proteobacteria*) [40]. Psychological stress reshapes microbiota through inflammatory and immune responses, often reducing beneficial bacteria like *Lactobacillus* and *Bifidobacterium* [41].

#### 2.3.5. Antibiotic Usage

Antibiotics significantly impact intestinal microbiota, reducing diversity and potentially allowing opportunistic pathogens, such as *C. difficile* [42,43], to proliferate. Antibiotic treatment typically disrupts bacteria like *Bacteroidetes, Firmicutes*, and *Actinobacteria*, which are critical for gut health [44]. Studies in infants show that antibiotics in early life can reduce beneficial bacteria (*Bifidobacterium*) and increase pathogens (*Klebsiella, Enterococcus*), with effects persisting up to a year [45]. Short-term use of antibiotics can lead to dysregulation of the bacteria, but the intestinal bacteria generally exhibit resilience through niche competition and immune regulation [46]. In contrast, long-term use of antibiotics significantly increases the proportion of resistant bacteria and promotes the spread of resistant genes. Resistant bacteria evolve under the pressure of antibiotic selection, acquiring stronger resistance through gene mutation and horizontal gene transfer. Prolonged antibiotic use not only alters the intestinal bacteria but also profoundly impacts the host’s immune system and metabolic function. Recovery of the intestinal bacteria after extended antibiotic use is more challenging and may not be fully achieved (Table 1).

## 3. Latest Research Progress

Intestinal bacteria are essential for human health, but when dysfunctional, they can harm the gut and other systems. Additionally, they influence the effectiveness of therapies like radiotherapy, chemotherapy, and immunotherapy and may serve as biomarkers to predict drug efficacy. Recent studies have highlighted the potential of fecal microbiota transplantation (FMT) for treatment, drawing attention to the mechanisms through which bacteria cause diseases and influence therapeutic outcomes. We review how bacterial composition, functions, and pathogenicity contribute to disease prevention and treatment.

### 3.1. Relationship Between Intestinal Bacteria and Gastrointestinal Diseases

#### 3.1.1. Inflammatory Bowel Disease (IBD)

IBD, encompassing Crohn’s disease (CD) and ulcerative colitis (UC), is a chronic, progressive gastrointestinal condition with unclear etiology. Studies [76] reveal reduced microbiome diversity in IBD patients, with fewer anti-inflammatory bacteria (such as *Bacteroidetes* and *Firmicutes,* especially *Clostridium*) and increased inflammatory bacteria. This imbalance, combined with oxidative stress, increases anaerobic and sulfate-reducing bacteria, which exert toxic effects on intestinal epithelial cells and may result from an abnormal mucosal immune response [77]. For instance, *Clostridium prevotellii*, normally fermenting non-digestible carbohydrates to produce SCFAs, is reduced in IBD, impacting Treg cell differentiation and intestinal epithelial growth [78]. Butyrate is a crucial anti-inflammatory factor for mucin synthesis in the gut. Conversely, adherent-invasive *Escherichia coli* (AIEC) increases sharply in IBD, impairing gut permeability and altering inflammatory cell regulation [3].

Autophagy also plays a role in IBD, with autophagy gene variants linked to CD [79]. Disrupted autophagy affects gut bacteria composition, contributing to disease progression. Additionally, altered glycosyltransferase activity changes glycan structures, disrupting the mucus layer and mucosal immunity, exacerbating IBD [80]. Bacterial metabolites, including bile acids, tryptophan, and succinic acid, are pivotal in IBD [80]. Bile acid malabsorption often accompanies IBD, with higher primary bile acids and lower secondary bile acids affecting immune pathways like FXR and TGR5 [81]. Tryptophan metabolites act as ligands for the aryl hydrocarbon receptor (AhR) [82], regulating immune responses and inflammation, where increased tryptophan degradation through the kynurenine pathway correlates inversely with disease activity.

#### 3.1.2. Colorectal Cancer (CRC)

**Figure 3:**
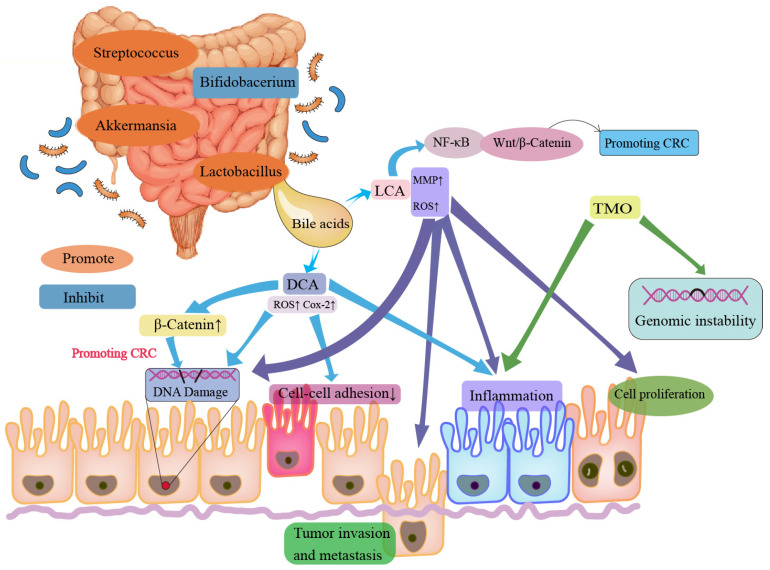
Promoting effects of intestinal bacterial metabolites on CRC. Metabolites, including bile acids (e.g., DCA and LCA) and trimethylamine n-oxide (TMAO), promote CRC development by increasing cancer cell proliferation, increasing DNA damage, enhancing tumor invasion and metastasis, reducing cell–cell adhesion, and promoting genomic instability.

Intestinal microbiota dysfunction plays a fundamental role in CRC development, as microbial imbalances damage the mucosal epithelium, influence host metabolism, and produce carcinogens, promoting tumorigenesis (Figure 3). Studies indicate distinct microbial profiles in CRC patients, with enrichments of bacteria such as *Fusobacterium nucleatum, Porphyromonas, Peptostreptococcus anaerobius*, and *Bacteroides fragilis*, which induce tumor proliferation, DNA damage, and immune evasion. Meanwhile, beneficial bacteria like *Faecalibacterium*, *Blautia*, *Lachnospira*, and *Bifidobacterium* are depleted in CRC, with tumor tissue often exhibiting reduced bacterial diversity compared to healthy tissue [83].

Intestinal bacteria promote CRC via multiple pathways, including metabolic byproducts, immune modulation, and inflammation. For instance, bile acid metabolites like cholic acid (CA) and chenodeoxycholic acid (CDCA) activate pathways such as NF-κB and JAK2/STAT3, contributing to CRC progression [84]. Primary bile acids are converted by bacteria into secondary forms like deoxycholic acid (DCA) and lithocholic acid (LCA), which increase cancer risk by promoting inflammatory responses and inhibiting DNA repair [85]. SCFAs, including butyrate, propionate, and acetate, protect against CRC by supporting cell homeostasis and immune responses. Butyrate, produced by Firmicutes [86], acts as a histone deacetylase (HDAC) inhibitor, encouraging apoptosis in CRC cells through Wnt/β-catenin pathway suppression, which bolsters the immune response [87]. Butyrate also inhibits angiogenesis and cell migration, while acetate can be converted into butyrate by specific bacteria, reinforcing these protective effects [88].

Intestinal microbiota also modulate immune responses within the tumor microenvironment (TME) by regulating inflammatory mediators and interacting with pattern recognition receptors (PRRs) [89]. For instance, E. coli upregulates COX-2 in macrophages, increasing PGE2 and promoting tumor growth [90], while Enterotoxigenic Fragile Bacillus (ETBF) activates NF-κB through IL-17 signaling, leading to immune cell accumulation in tumors [56]. Similarly, Fusobacterium nucleatum in CRC patients activates Wnt/β-catenin signaling, increasing inflammatory cytokines (e.g., IL-6, IL-8) and fostering tumor proliferation [91].

#### 3.1.3. Gastric Cancer (GC)

*Helicobacter pylori* infection is the primary risk factor for GC, with virulence factors like the Cag Pathogenic Island (cag PAI) and CagA protein altering host signaling pathways [92]. *H. pylori* suppress tumor suppressors (e.g., p14ARF [68], USF1 [69]), disrupt the gastric mucosal barrier, and drive epithelial–mesenchymal transition (EMT), fostering GC development [93]. The TME surrounding tumor cells, comprising immune cells, fibroblasts, and signaling molecules, significantly influences GC progression [94]. For example, IL-6 in the TME activates JAK/STAT, promoting tumor growth, while pathways like MEK/ERK maintain tumor integrity. *H. pylori* also activate gastric fibroblasts, encouraging them to secrete factors like TGF-β, which support EMT and tumor invasion [95].

However, *H. pylori* alone accounts for only a small percentage of GC cases, as other intestinal bacteria likely contribute [96]. Studies revealed that GC patients often have increased levels of *Lactobacillus, Fusobacterium, Veillonella*, and *Haemophilus*, among others, which may synergize with *H. pylori* to promote tumor progression [97]. For instance, *H. pylori* and *Bacteroides fragilis* both produce H_2_O_2_ during polyamine oxidation, adding to oxidative stress in gastric tissues and advancing cancer [98].

#### 3.1.4. Irritable Bowel Syndrome (IBS)

IBS pathogenesis involves intestinal inflammation, barrier dysfunction, and altered gut–brain interactions, now seen as a microbiota–gut–brain axis disorder. In IBS patients, intestinal dysbiosis, characterized by an increased *Firmicutes/Bacteroidetes* ratio, promotes pathogen adhesion to the mucosa and drives inflammation. Normally, the vagus nerve modulates gut responses to microbial metabolites like SCFAs, bile acids, and neurotransmitters (e.g., serotonin and dopamine), all of which are altered in IBS [99,100]. Changes in bile acid synthesis, mediated by gut bacteria and enzymes like CYP7A1, can affect pain perception and bowel function [101].

In IBS, SCFA levels vary by subtype: patients with diarrhea-predominant IBS have higher fecal SCFAs, while those with constipation-predominant IBS have lower SCFA levels [102,103]. This correlation between SCFA concentration and symptom severity suggests microbial metabolites contribute to IBS symptoms through mechanisms like GPCR activation and epigenetic effects, influencing inflammation, metabolism, and mucosal integrity. For example, tryptophan metabolites impact IBS via AhR signaling [104], and intestinal bacteria regulate 5-HT (serotonin) levels by inducing tryptophan hydroxylase [105], impacting holobiont motility and homeostasis. Additionally, bacterial metabolites trigger neuropeptide secretion (e.g., GLP-1 [106], PYY [107]), influencing bowel transit. Interestingly, GLP-1 levels are lower in constipation-type IBS than in diarrhea-type IBS, suggesting gut bacteria differently modulate symptoms in these subtypes [108].

### 3.2. Relationship Between Intestinal Bacteria and Other Diseases

The influence of intestinal bacteria extends well beyond digestion, affecting systemic health by contributing to various disease states. Significant changes in microbial composition and function are associated with cardiovascular, metabolic, autoimmune, and neuropsychiatric diseases [109].

#### 3.2.1. Cardiovascular Disease (CVD)

Early sequencing studies, such as those by Koren et al. [110], identified bacterial DNA within human atherosclerotic plaques, suggesting an association between intestinal bacteria and CVD. A key metabolite, trimethylamine N-oxide (TMAO), produced after gut bacteria metabolize Western diet nutrients, has been shown to promote atherosclerosis and thrombosis [111]. TMAO enhances cholesterol accumulation, promotes platelet activation, and induces vascular inflammation through pathways like MAPK and NF-κB, resulting in increased inflammatory gene expression [112]. In preclinical models, oral administration of *Akkermansia muciniphila* in ApoE knockout mice reduced intestinal permeability and LPS levels, leading to reduced aortic atherosclerosis [113]. Human studies similarly observed that A. muciniphila reduced plasma LPS levels in metabolic syndrome patients, mitigating inflammation and lowering CVD risk. Additionally, acetate and propionate influence blood pressure by activating GPCRs, such as Olfr78 and Gpr41, which help regulate vascular resistance and renin release [114].

#### 3.2.2. Metabolic Disease

Intestinal dysbiosis is strongly linked to metabolic diseases, notably obesity and metabolic syndrome, characterized by dyslipidemia, glucose intolerance, and hypertension. Genomic studies show that obese individuals tend to have a higher *Firmicutes/Bacteroidetes* ratio, which enables greater calorie extraction from food [115]. Reduced levels of beneficial bacteria, including *Akkermansia*, *Faecalibacterium*, *Oscillibacter*, and *Alistipes*, are also observed [116]. Obesity-associated metabolites, such as LPS, increase gut permeability, leading to endotoxemia and insulin resistance [117]. LPS triggers inflammation by binding to Toll-like receptor 4 (TLR4), a pathway that is upregulated in obese and type 2 diabetes patients [118]. Additionally, specific bacterial metabolites, including imidazole propionate, branched-chain amino acids, and SCFAs, can disrupt insulin signaling and glucose metabolism, contributing to diabetes and obesity [119].

#### 3.2.3. Autoimmune Diseases (ADs)

Autoimmune diseases (ADs) arise when the immune system mistakenly attacks the body’s tissues. Although AD pathogenesis is complex, involving genetic, environmental, and microbial factors, growing evidence suggests that changes in intestinal bacteria composition influence disease risk [120]. For example, patients with systemic lupus erythematosus (SLE) and type 1 diabetes (T1D) show a lower Firmicutes/Bacteroidetes ratio [121]. In multiple sclerosis (MS) patients, increased levels of *Methanobrevibacter* and *Akkermansia* and reduced *Butyricimonas* are observed [122]. In rheumatoid arthritis (RA), reduced *Faecalibacterium* and increased *Eggerthella* and *Collinsella* have been reported [123]. Some bacteria, including *Lactobacillus acidophilus* and *Bifidobacterium*, enhance gut immune function by increasing Treg cells, reducing Th1 responses, and enhancing gut barrier integrity, thereby offering protection against T1D [124]. Studies also link higher α-diversity with milder disease progression in Chinese RA patients [125]. Certain oral bacteria, such as *Porphyromonas gingivalis*, may contribute to RA by migrating to the gut, disrupting microbiota, increasing permeability, and promoting the production of anti-citrullinated protein antibodies (ACPA) [126].

#### 3.2.4. Neuropsychiatric and Neurological Diseases

Intestinal bacteria influence neuropsychiatric and neurological disorders primarily through stress and inflammation pathways. Psychological stress affects conditions like depression, schizophrenia [127], autism spectrum disorder (ASD), epilepsy, and migraine, while inflammation is linked to Parkinson’s disease (PD), schizophrenia, and migraine. Studies indicate that depression patients have elevated serum IgM and IgA against *Enterobacter* LPS, suggesting “leaky gut” may play a role [128]. Patients with depression also exhibit increased *Enterobacteriaceae* and *Alistipes* and decreased *Faecalibacterium* [129]. In ASD, reduced populations of *Prevotella, Coprococcus, Bacteroides,* and *Actinomyces* and increased *Desulfovibrio* are observed [130]. In PD, *Faecalibacterium* and *Lachnospiraceae* levels are reduced, while *Bifidobacteriaceae* and *Ruminococcaceae* are elevated [131]. Dysregulated bacteria release pro-inflammatory cytokines (e.g., TNF-α, IL-1β, IL-6), exacerbating pain pathways in conditions like migraine and impacting the gut–brain axis in neurodegenerative diseases [132].

### 3.3. Latest Testing and Diagnostic Methods

Intestinal bacteria are deeply involved in the onset and progression of numerous human diseases. Bacterial metabolites enter the circulatory system, affecting remote organs, while bacterial genes and proteins are detectable with advanced technologies, supporting disease diagnosis, treatment, and prognosis.

16S rRNA gene sequencing is an accessible, affordable next-generation sequencing (NGS) platform that provides insight into the taxonomic composition of a sample. 16S rRNA gene sequencing is typically accurate only at the genus level, making it difficult to distinguish between different species or strains within the same genus, and it cannot directly reflect microbial gene function or metabolic activities. Therefore, it has limitations in studying the functional association between microorganisms and diseases. In contrast, whole-genome shotgun sequencing maps taxonomic distribution at the species and even strain level and assesses genes, including those involved in metabolic pathways. Though interstudy consistency in shotgun metagenomics remains a challenge, this method improves functional characterization across microbiome studies, helping researchers identify specific microbial genomes, including novel, unnamed species, from sequence read pools [133]. However, it offers more detailed insights but requires higher sequencing depth and more complex bioinformatic analysis, significantly increasing costs.

Metagenomic sequencing enables the characterization of microbial activity and functionality and links metagenomes to downstream metabolomic and proteomic profiles. In colorectal cancer (CRC) and adenoma patients, a study of 885 altered serum metabolites identified eight gut bacteria-related serum metabolites (GMSM panel) that could accurately differentiate CRC and adenoma patients from healthy individuals. These findings suggest that intestinal microbiota reprogramming in CRC is reflected in the serum metabolome, where GMSM changes offer a noninvasive diagnostic marker [134]. Serum metabolites levels, affected by numerous factors, may lead to large individual variations, affecting diagnosis accuracy and requiring multiple samples for reliable results, further increasing testing complexity and cost. Additionally, metabolites may be associated with multiple diseases rather than specifically reflecting changes in the intestinal bacteria, which can lead to misdiagnosis.

Fecal microbial markers show strong potential as diagnostic tools, being accurate, economical, and noninvasive [135]. Specific bacterial species such as Fusobacterium, Bifidobacterium [136], and colibactin-producing bacteria [137] can enhance the sensitivity and specificity of traditional fecal immunochemical tests (FIT), offering promising applications in CRC screening. Serum antibody tests for certain bacteria, like Fusobacterium, have shown diagnostic potential in CRC [138], with intestinal bacterial metabolites also serving as indicators for colorectal adenoma prognosis and CRC screening [139]. FIT can be affected by diet and medications, causing false positives. While these tests are valuable for disease diagnosis and screening, their limitations should be considered to enhance diagnosis accuracy and reliability.

### 3.4. Anti-Cancer Therapy

By regulating the composition and function of the gut microbiota, immune response, metabolic function, nerve signaling, and inhibition of pathogen growth, intestinal bacteria play a crucial role in treating relative diseases. In non-cancer diseases, probiotic supplementation, FMT, and dietary interventions are common therapeutic strategies, but their specific applications need to be tailored to disease type and the patient’s individual situation. This review mainly focuses on the role and significance of intestinal bacteria in anti-cancer therapy.

#### 3.4.1. Chemotherapy

Intestinal bacteria regulate chemotherapy responses via immune modulation, translocation, and enzymatic activity. Chemotherapeutics alter the tumor microenvironment (TME) through interactions with intestinal bacteria, enhancing tumor-targeting immune responses (Table 2) [140]. For example, platinum drugs like oxaliplatin and cisplatin induce cytotoxicity through platinum–DNA adducts, and lack of intestinal bacteria reduces the production of ROS and subsequent drug efficacy [141]. *Fusobacterium nucleatum* is associated with chemotherapy resistance by promoting autophagy [142], while the anti-tumor efficacy of cyclophosphamide (CTX) is diminished when broad-spectrum antibiotics reduce intestinal bacteria [143]. CTX mediates anti-tumor immune responses through specific Gram-positive bacteria that stimulate subsets of pathogenic T-helper cells, promoting the differentiation of immature T cells [144]. Additionally, certain Gram-negative bacteria enhance CTX’s effects by affecting T cell recruitment, highlighting intestinal microbiota as a target to improve chemotherapy outcomes.

#### 3.4.2. Radiotherapy

Radiotherapy alters the composition of intestinal bacteria and their metabolites, potentially influencing patient response to treatment [153]. Microbiota-regulated protein FIAF is associated with the radiosensitivity of cells and can protect against radiotoxicity. While *Bacteroides thetaiotaomicron* and *Enterococcus faecalis* increase FIAF production, *E.coli* reduces it [154]. Although human and mouse studies suggest that certain bacteria are associated with radiation-induced toxicity (e.g., *Bacilli*, *Lachnospiraceae*, *Akkermansia*, *Faecalibacterium*), a clear understanding of intestinal bacteria’s effect on radiotherapy efficacy remains elusive, warranting further research [155].

#### 3.4.3. Immunotherapy

Immunotherapies, notably immune checkpoint inhibitors (ICIs), treat solid tumors by enhancing T cell activation and blocking inhibitory receptors like PD-1 or CTLA-4, strengthening immune attacks on tumor cells [156]. Specific bacteria, including *Akkermansia muciniphila, Bifidobacterium,* and *Lactobacillus,* enhance immunotherapy effectiveness through anti-tumor metabolites like SCFAs [151]. In CRC mice treated with anti-CTLA-4 and Lactobacillus acidophilus lysates, tumor reduction was associated with increased IL-2, IFN-γ, and CD8+ T cell infiltration, with Bifidobacterium upregulating tumor-specific CD8+ T cells and IFN-γ secretion [157]. Analysis has shown that patients responding to anti-PD-1 therapy display higher bacterial diversity and α-diversity, with Faecalibacterium enriched in responders and *Bacteroides* and *Escherichia* in non-responders [152]. Intestinal microbiota modulation may enhance immunotherapy in immunogenically poor tumors by improving immune cell infiltration and activating key immune responses.

#### 3.4.4. Probiotics, Prebiotics, and Postbiotics

Probiotics, prebiotics, and postbiotics play critical roles in cancer prevention and management, with mechanisms including inflammation inhibition, early tumor cell apoptosis, and gut barrier restoration. Probiotics reduce chemotherapy- and radiotherapy-induced mucositis by preserving gut barrier integrity, reducing inflammation, and inhibiting apoptosis, supporting the idea that holobiont interventions can optimize cancer therapies [158].

### 3.5. Fecal Microbiota Transplantation (FMT)

FMT transfers gut bacteria from a healthy donor into a recipient’s digestive tract to restore microbial balance, a procedure already used to treat recurrent *Clostridium difficile* infections (rCDI). In animal models, FMT from healthy donors has significantly improved chemoradiotherapy outcomes by restoring microbial balance and reducing gastrointestinal toxicity [159]. FMT administration methods include nasogastric tube, oral capsule [160], colonoscopy, and enema [161], with oral capsules showing the highest cure rates and comfort levels.

FMT shows promise in treating disorders like IBS and hepatic encephalopathy (HE), though further research is needed to confirm efficacy across conditions [162]. For graft-versus-host disease (GVHD), FMT may reduce treatment resistance, improving patient survival [162]. Additionally, FMT has shown potential in reducing insulin resistance in metabolic syndrome [163], alleviating autism spectrum disorder (ASD) symptoms [164], and protecting against neuroinflammation in Parkinson’s disease (PD) [165]. Studies indicate FMT’s potential benefits in diseases requiring microbiota reset, such as microscopic colitis, celiac disease, and constipation, though more research is needed to develop standardized protocols and confirm efficacy in various diseases.

## 4. Conclusions and Perspectives

This review emphasizes the role of intestinal bacteria in disease pathogenesis and recent research advancements. These findings are essential for improving diagnostic and therapeutic strategies. Clinicians should consider patient-specific factors when developing treatment plans and collaborate with multidisciplinary teams, including microbiologists and nutritionists, to address complex clinical challenges. In recent years, the interactions in holobiont in disease processes have gained significant attention, with many studies demonstrating their potential not only in treating gastrointestinal diseases but also in addressing conditions affecting other systems by targeting the gut microbiome. However, despite the promise of bacterial analysis in diagnosing these diseases, several challenges remain. Large-scale clinical studies are still needed to confirm the accuracy and reliability of bacterial biomarkers. Additionally, individual variations in gut bacteria composition must be taken into account, as these differences can influence how effective bacteria may be as diagnostic markers.

## Figures and Tables

**Table 1:** Classification and functions of intestinal bacteria in human body and the effects of disease and pathogenic mechanisms.

Classification	Bacteria	Functions	Effect in Diseases	Mechanisms
*Firmicutes*	*Clostridium* *butyricum*	Produce SCFAs Regulate immunity Protect intestinal barrier Modulate intestinal bacteria	Inhibit	Decrease secondary BAs secretion Inhibit proliferation of CRC cells [47] Induce apoptosis in CRC cells [47] Inhibit Wnt/β-catenin signaling pathway [47] Activate GPRs including GPR43 and GPR109A [48]
	*Streptococcus* *gallolyticus*	Produce SCFAs Regulate immunity	Promote	Activate NF-κB andlL-8 [49] Inflammatory reaction Proliferate CRC cells through β-catenin but not inflammation
	*Streptococcus* *thermophilus*	Produce SCFAs Regulate immunity Protect intestinal barrier	Inhibit	Inhibit the invasion of Escherichia coli Product β-Galactosidase to enhance abundance of probiotics including Bifidobacterium and Lactobacillus [50] Activate oxidative phosphorylation Express intestinal homing receptor CCR9 to against T1D
	*Lacticaseibacillus* *paracasei*	Synthesis vitamins Protect intestinal barrier	Inhibit	Inhibit proliferation of CRC cells Induce apoptosis of CRC cells Lactobacillus paracasei DTA81 diminish liver oxidative [51] stress and decrease proliferating cell nuclear antigen
	*Ruminococcus* *gnavus*	Produce SCFAs Regulate immunity Protect intestinal barrier	Inhibit	Degrade lysoglycerophospholipid [52] Promote tumor immune surveillance function of CD8+T cell [52]
	*Lactobacillus*	Synthesis vitamins (especially VB12) Inhibit pathogens Protect intestinal barrier Regulate immunity	Inhibit	Stimulate antimicrobial peptides and sIgA to improve tight junction integrity [53,54] Enhance the levels of IgA, IgG and IgM Promote T and B lymphocyte proliferation to strengthen immunity [54] Stimulate the secretion of ROS, lysosomal enzymes and mononuclear factors to enhance non-specific immune response [54] Express intestinal homing receptor CCR9 to against T1D [55]
*Bacteroidetes*	*Bacteroides* *fragilis*	Synthesis vitamin K Regulate immunity	Promote	Interaction with lL-17 [56] Activate NF-κB [56] Accumulate regulatory T cells to enhance inflammatory response Activate β-catenin signaling pathway [57] Proliferate CRC cells DNA damage through polyamine metabolism [58] Induce bacterial dysbiosis by promoting procarcinogenic bacteria [59] Disturb the host immune system and intestinal barrier Promote mucin degradation
	*Enterotoxigenic* *Bacteriodes fragilis*	Secrete BFT Regulate immune system	Promote	Activate Stat3 pathway via BFT [60] Activate NF-κB signaling pathway via IL-17R [61] Trigger chemokines expression to promote the accumulation of myeloid cells [62]
*Actinobacteria*	*Bifidobacterium*	Produce SCFAs Synthesis essential vitamins (folic acid and vitamin K) Inhibit pathogens Protect intestinal barrier	Inhibit	Promote tumor immune surveillance function of CD8+T cell Affect metabolism of intestinal probiotics to reduce carcinogens [63] Induce cell apoptosis to prevent the development of tumors [64] Express intestinal homing receptor CCR9 to against T1D [55]
*Proteobacteria*	*Escherichia coli*	Regulate immunity	Promote	Inflammatory reactions [65] DNA damage Secrete virulence factors to activate COX-2 Promote cell-cycle arrest [66] Induce CRC cells apoptosis [66] Disrupt the intestinal vascular barrier
	*Helicobacter pylori*	Secrete cytotoxins such as VacA, CagA and urease	Promote	Inject toxins into host cells and affect signal transduction pathways [67] Inhibit the expression of tumor suppressor p14ARF and transcription factor USF1 [68,69] Disrupt gastric mucosal barrier Activate GCAFs to promote the development of EMT and GC by altering TME [70] Promote oxidative stress to lead malignant tumor [71]
*Fusobacteria*	*Fusobacterium* *nucleatum*	Regulate immunity Secrete FadA	Promote	Promote proliferation of tumor cells [72] DNA damage and carcinogenesis through ROS Mutate in the Apc tumor suppressor gene [73] Increase tumor-promoting cytokines through NF-*κ*B activation Secrete FadA adhesion protein activating Wnt/*β*-catenin signaling Inhibit T cell activation and NK cell-meditated killing of tumor [74] Suppress immune surveillance
*Verrucomicrobacteria*	*Akkermansia muciniphila*	Produce SCFAs Protect intestinal barrier Synthesis vitamins (especially VB12) Regulate immunity	Inhibit	Reduce LPS to inhibit pro-inflammatory cytokines by binding to TLRS and decrease the risk of CVD [75] Produce SCFAs to lower blood pressure through GPCRs such as Olfr78 and Gpr41

Abbreviation: CRC: colorectal cancer; SCFAs: short-chain fat acids; BAs: bile acids; GPR: G-protein coupled receptor; NF-κB: nuclear factor-kappa B; CCR9: chemokine receptor 9; T1D: type 1 diabetes; sIgA: secreted immunoglobulin A; ROS: reactive oxygen species; BFT: *Bacteroides fragilis* toxin; COX-2: cycloocygenase-2; p14ARF: p14 alternate reading frame; USF1: upstream transcription factor 1; GCAFs: gastric cancer-associated fibroblasts; EMT: epithelial–mesenchymal transformation; VacA: vacuolating cytotoxin; CagA: cytotoxin-associated Protein; GC: gastric cancer; TME: tumor microenvironment; FadA: Fusobacterium adhesin A; NK: natural killer; LPS: lipopolysaccharide; TLRs: toll-like receptors; CVD: cardiovascular disease.

**Table 2:** Effects and roles of different intestinal bacteria on chemotherapy effect and their mechanisms.

Species	Roles	Effects	Mechanisms
*Fusobacterium nucleatum*	Beneficial	Enhance anti-PD-1 therapy efficacy	Activate STING signaling; recruit IFNγ+ CD8+ tumor infiltrating lymphocytes [145]
*Lactobacillus*	Beneficial	Increase the sensitivity to 5-FU or alleviate FOLFOX-induced mucosal damage	Promote SCAFs tumor suppression [146] or down-regulate NF-κΒ pathway, TNF-α and IL-6 [147]
*Streptococcus*	Beneficial	Alleviate irinotecan-induced diarrhea and toxicity	Not available
*Bacteroides*	Beneficial	Increase FIAF (a protective agent against radiotoxicity); enhance 5-FU anti-CTLA-4 therapy efficacy	Activate immune response [148]
*Banethera enterobanethis*	Beneficia	Enhance CTX efficacy	Increase the recruitment or proliferation of IFN-c+cdT cells in tumor-infiltrating lymphocytes [149]
*Bifidobacterium*	Beneficial	Enhance 5-FU and anti-PD-1 therapy efficacy	Promote SCAFs tumor suppression, DC maturation and CD8+ T cell activation [150]
*Akkermansia muciniphila*	Beneficial	Enhance anti-PD-1 therapy efficacy	Promote SCAFs tumor suppression [151]
*Faecalibacterium*	Beneficial	Enhance anti-PD-1 therapy efficacy	Enhance the level of CD8+ TIL [152]
*Enterococcus faecalis*	Beneficial	Increase FIAF production	Not available
*Fusobacterium nucleatum*	Detrimental	Promote 5-FU and oxaliplatin resistance	Activate TLR4/MYD88 to induce autophagy [142]
*Escherichia coli*	Detrimental	Decrease FIAF production	Not available

Abbreviation: PD-1: programmed cell death-1; 5-FU: 5-fluorouracil; STING: stimulator of interferon genes; CTLA-4: cytotoxic T lymphocyte-associated antigen-4; CTX: cyclophosphamide; IFN: interferon; MYD88: myeloid differentiation primary response88.
